# Forecasting Heliospheric CME Solar-Wind Parameters Using the UCSD Time-Dependent Tomography and ISEE Interplanetary Scintillation Data: The 10 March 2022 CME

**DOI:** 10.1007/s11207-023-02169-8

**Published:** 2023-05-30

**Authors:** Bernard V. Jackson, Munetoshi Tokumaru, Kazumasa Iwai, Matthew T. Bracamontes, Andrew Buffington, Ken’ichi Fujiki, Go Murakami, Daniel Heyner, Beatriz Sanchez-Cano, Mathias Rojo, Sae Aizawa, Nicolas Andre, Alain Barthe, Emmanuel Penou, Andrei Fedorov, Jean-Andre Sauvaud, Shoichiro Yokota, Yoshifumi Saito

**Affiliations:** 1 Center for Astrophysics and Space Sciences, University of California, San Diego 0424, La Jolla, CA 92093-0424 USA; 2 Institute for Space-Earth, Environmental Research, Nagoya University, Furo-cho, Chikusa-ku, Nagoya, 464-8601 Japan; 3 Japan Aerospace Exploration Agency, Sagamihara, Kanagawa 299-8510 Japan; 4 Institute for Geophysics and Extraterrestrial Physics, Braunschweig, Germany; 5 School of Physics and Astronomy, University of Leicester, Leicester, United Kingdom; 6 IRAP, CNRS-UPS-CNES, Toulouse, France; 7 Department of Physics, University of Pisa, Pisa, Italy; 8 Osaka University, Osaka, Japan

**Keywords:** Coronal Mass Ejections (CMEs), Interplanetary Scintillation (IPS), Institute for Space-Earth Environmental Research (ISEE), Three-dimensional (3D) reconstructions, Solar wind, Inner heliosphere

## Abstract

Remotely sensed interplanetary scintillation (IPS) data from the Institute for Space-Earth Environmental Research (ISEE), Japan, allows a determination of solar-wind parameters throughout the inner heliosphere. We show the 3D analysis technique developed for these data sets that forecast plasma velocity, density, and component magnetic fields at Earth, as well at the other inner heliospheric planets and spacecraft. One excellent coronal mass ejection (CME) example that occurred on the 10 March 2022 was viewed not only in the ISEE IPS analyses, but also by the spacecraft near Earth that measured the CME arrival at one AU. Solar Orbiter, that was nearly aligned along the Earth radial at 0.45 AU, also measured the CME in plasma density, velocity, and magnetic field. BepiColombo at 0.42 AU was also aligned with the STEREO A spacecraft, and viewed this CME. The instruments used here from BepiColombo include: 1) the European-Space-Agency Mercury-Planetary-Orbiter magnetic field measurements; 2) the Japan Aerospace Exploration Agency Mio spacecraft Solar Particle Monitor that viewed the CME Forbush decrease, and the Mercury Plasma Experiment/Mercury Electron Analyzer instruments that measured particles and solar-wind density from below the spacecraft protective sunshield covering. This article summarizes the analysis using ISEE, Japan real-time data for these forecasts: it provides a synopsis of the results and confirmation of the CME event morphology after its arrival, and discusses how future IPS analyses can augment these results.

## Introduction

**Figure 1:**
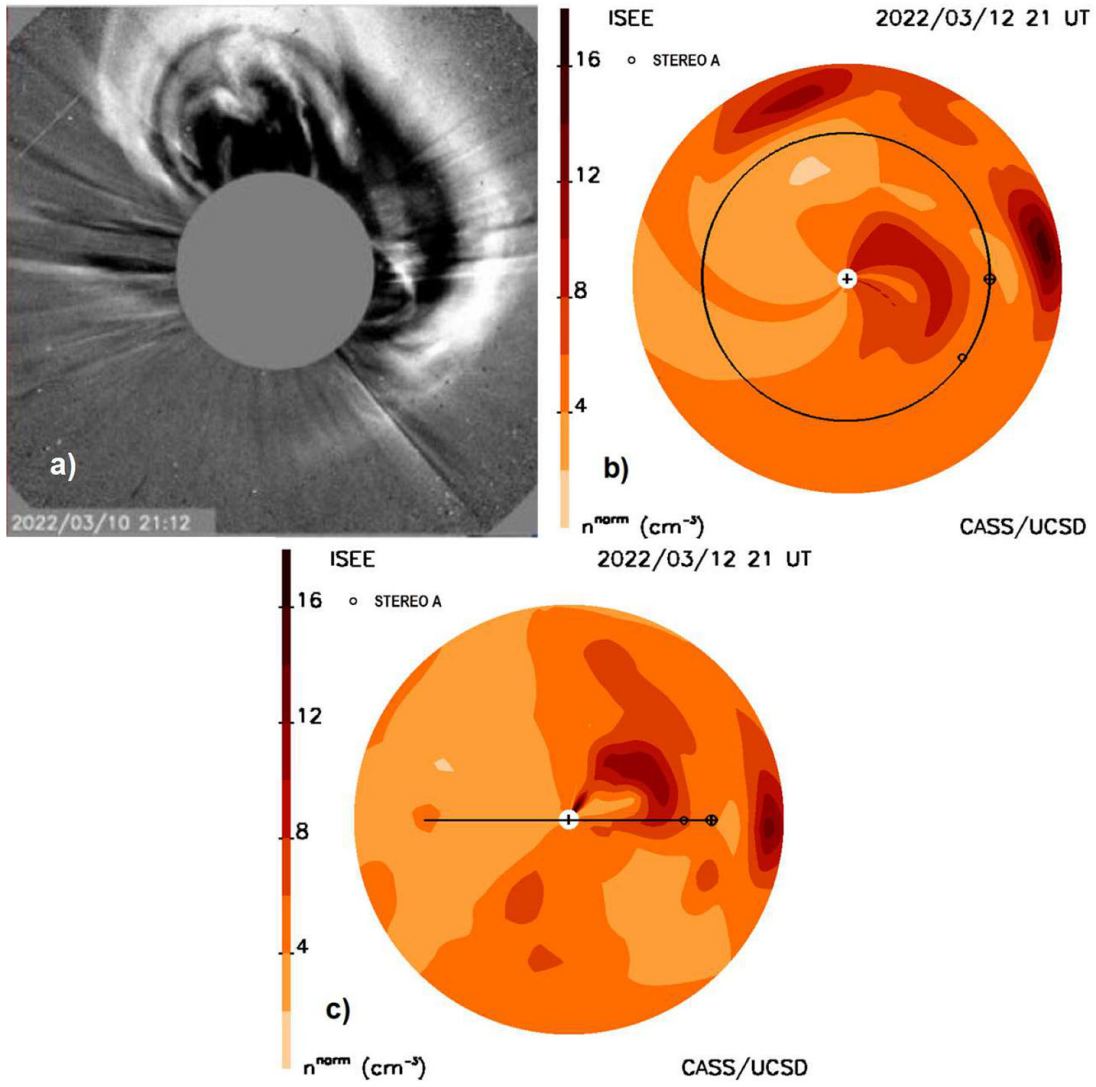
**a**) The 10 March 2022 halo CME as observed by the SOHO C2 coronagraph. **b**) The CME as observed using a time-dependent fitted model to IPS g-level data presented as a density ecliptic cut two days later at 21 UT March 12, 2022. The Earth is superposed on its orbit to the right, with STEREO A positioned to the lower right. An r^−2^ density fall-off has been removed from the volumetric data to better display structures both near and far from the Sun. **c**) A density meridional cut through Earth showing the CME structure at the same time as in b). Earth, its orbit, and the STEREO spacecraft locations are projected onto this plane.

The current state-of-the-art in interplanetary scintillation (IPS) Coronal Mass Ejection (CME) forecasting analysis is shown in Figure 1. In the late 1950s Parker (1958) postulated that the solar wind became supersonic near the Sun and thus, the solar wind contained plasma structure that traveled the distance from Sun to Earth over several days. Neugebauer and Snyder (1962) presented measurements of solar-wind speed from the Mariner 2 spacecraft enroute to Venus that showed the high speed of the solar wind was a valid concept. During the same period, radio telescopes were designed with high-enough spatial resolution, signal-to-noise, and a short cadence between observations to enable discerning the intensity scintillation of point-like radio sources (Ryle and Neville, 1962). These interplanetary scintillation (IPS) observations were used to sense the solar wind between Sun and Earth (Clarke, 1964; Hewish, Scott, and Wills, 1964), and provide additional remote views of its outward flow speed. Known to arise from small-scale electron-density inhomogeneities in the solar wind, on the order of a few tens to hundreds of km in size, it was also clear that these could determine different types of large-scale solar-wind structures and its overall variable outward flow (Houminer, 1971) including CMEs (Jackson, 1982). Since the inner heliosphere could be observed prior to its Earth-arrival, it was also clear from the first observations that IPS had the potential to forecast the onset of CMEs and the geomagnetic storms they cause. These storms were often present when high levels of interplanetary scintillation engulfed the Earth.

However, it was not until the advent of large computer systems that the coordinate conversions and analyses needed to 3D reconstruct solar-wind structures quickly for forecasts became an amenable reality (Jackson et al., 1998; Kojima et al., 1998). The radio systems used at this early stage still exist, and were primarily designed to study the scintillation process itself, and slowly changing heliospheric structure. More recent IPS systems generally provide many purpose analyses, for both short-duration scientific solar and ionospheric studies, as well as low-frequency astrophysical studies. So far, the Institute for Space-Earth Environmental Research (ISEE), Nagoya University radio-telescope facility, and a few dedicated scientists and staff members are exceptions to this; for nearly a quarter of a century, they have provided their IPS data for public use in near real time on their FTP website.

**Figure 2:**
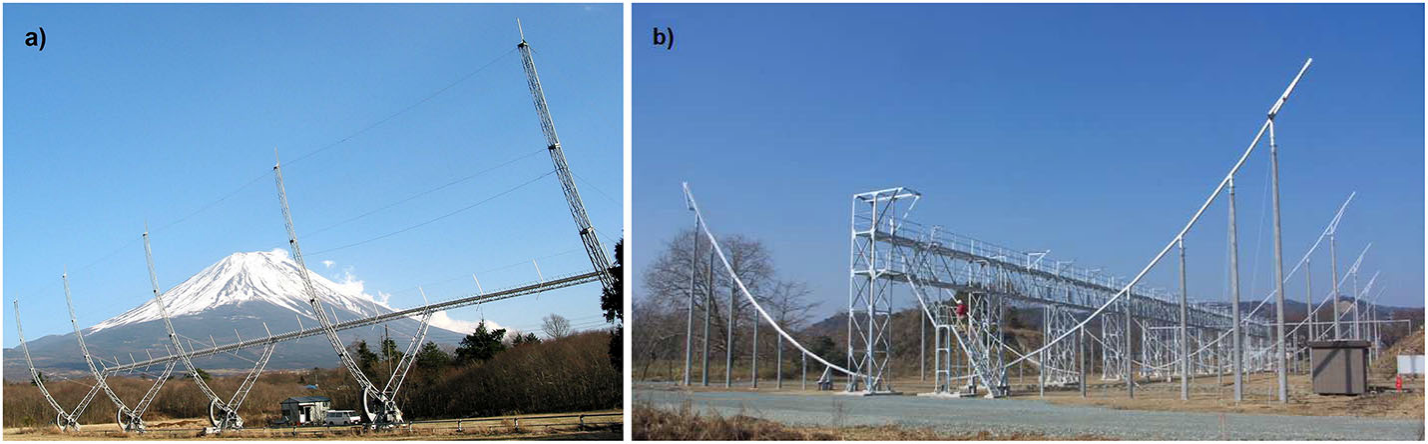
**a**) ISEE IPS radio array near Mt. Fuji. The arrays used singly measure scintillation intensity (or $g$-level). Scintillation signals crosscorrelated between arrays give a robust sky plane IPS velocity determination. **b**) A larger system in operation since 2010 measures ${g}$-levels at the Toyokawa radio site and integrates its results with the other telescopes.

Figure 2 shows two of the systems that are situated around central Japan. These have long been portions of the only system providing long-term measurement of variable solar-wind speed throughout several solar cycles, both in the ecliptic and over the solar poles (Tokumaru, Kojima, and Fujiki, 2012; Tokumaru, Fujiki, and Iju, 2015; Tokumaru et al., 2017). The scintillation data, too abundant to edit using “by hand” data-reduction techniques, has, since the early 1980s, been reduced by computer-analysis techniques (Kojima and Kakinuma, 1987), and presented on a website. ISEE staff have continued to refine their IPS radio system since its beginning, and have maintained their system with many upgrades through to the present. Since 2010 the ISEE system has provided year-round observations in scintillation level (Tokumaru et al., 2011). IPS velocities are also available from these data for most of the year. The solar-heliospheric group at the University of California at San Diego (UCSD) has used this data set since the year 2000 to provide an IPS time-dependent 3D reconstruction of heliospheric structures (Jackson et al., 2003), and with embellishments, has continued these analyses at: ips.ucsd.edu/ and at different associated websites. Now, at the beginning of Solar Cycle 25, these analyses can provide excellent plasma density, velocity, and component magnetic-field measurements for many heliospheric structures including CMEs in advance of their arrival at Earth. Now, too, there are many in-situ monitors near Earth and in the inner heliosphere that show how well these transient structures have been reconstructed.

In this article, we examine a halo CME in detail that erupted from the Sun on 10 March 2022, both to determine how well it was forecast by the UCSD tomographic analysis, and from retrospective measurements, how well it was reconstructed in 3D using the ISEE IPS data. Many of the current CME analyses use near-solar inputs and then project these outward through the heliosphere with little intervening heliospheric information to determine how well these modeling efforts work until Earth is impacted. The 10 March CME is well documented at the NASA Community Coordinated Modeling Center (CCMC) using the most prevalent technique (CME cone model - ENLIL 3D MHD modeling) which provides a scoreboard of forecast shock-arrival times at: kauai.ccmc.gsfc.nasa.gov/CMEscoreboard/PreviousPredictions/2022. For this CME, the scoreboard documents 13 forecasts that provide arrival times from two hours prior to nearly 15 hours past the actual arrival time. Now, from these spacecraft that provide intervening heliospheric information, the existing remotely sensed heliospheric information that provides the morphology and timing of CMEs, can also be revised.

The IPS 3D reconstruction technique is provided by a simple model: this assumes no shape for the structure other than that it flows outward radially, and that the material within the structure conserves mass and mass flux during this outward motion. The material within the structure is also assumed to vary smoothly through the remotely sensed line-of-sight (LoS) locations using known LoS weighting (Young, 1971) that provides fits to this model. Section 2 briefly describes the 3D reconstruction technique used. Section 3 shows the forecast analysis as it was obtained from the UCSD website prior to the 10 March 2022 CME arrival. Section 4 provides more forecast details of the CME morphology and analyses from additional data sets that have certified the validity of this global forecast. Section 5 describes how the 3D reconstruction details of the 10 March CME might have been even better fit at Earth by using higher resolution, a lower data latency, and more IPS data. We conclude in Section 6.

## The UCSD 3D Reconstruction Technique

The UCSD time-dependent 3D reconstruction determines heliospheric structure and predicts both CME and corotating structure arrival at Earth (Jackson and Hick, 2005; Jian et al., 2015, 2016; Gonzi et al., 2020). Jackson et al. (2020) present a further review of the technique and its background. The analysis was designed initially as a purely corotational technique (see Kojima et al., 1998; Jackson et al., 1998), but for UCSD this was modified in the year 2000 to a time-dependent form. The mathematical details of the corotating IPS 3D reconstruction technique are given in Hick and Jackson (2004), and the IPS time-dependent technique for IPS in Jackson et al. (2008a). More details of the UCSD IPS kinematic model near-real-time forecasting analysis are given in Jackson et al. (2010, 2011a, 2013). The 3D reconstruction proceeds by least-squares fitting a kinematic heliospheric solar-wind model to observations along the IPS lines-of-sight (also LoS). The appropriate LoS weighting for IPS is derived from theoretical weak scattering theory (Young, 1971; Kojima et al., 1998). This, and the density along the LoS, gives the modeled value of IPS normalized scintillation strength ($g$-level), to compare with observed values (see Jackson et al., 2020, 2022). To ensure the tomographic $g$-level values at Earth agree with near-Earth in-situ measurements, the mean proton-density value of the time series, where in-situ data are available, are compared with the average IPS $g$-level values at Earth to set them to a value of 1.0 for a two-week period prior to the analysis run time. This average value, generally ∼5 Np (cm^−3^), provides the background density as a standard value that varies throughout the inner heliosphere to fit the LoS IPS $g$-level measurements. Over the period prior to the run time there are usually fewer than 100 line-of-sight IPS values available each day; this limits the 3D reconstructions to a cadence of one day, and a latitude and longitude resolution of 20° × 20°. The data are then interpolated from each daily source surface for in-situ comparisons and as volumetric data with a 6-hour cadence and a three-times higher latitude–longitude resolution. For more details about this fitting technique see Jackson et al. (2013, 2020, 2022), and Tiburzi et al. (2022), where the confirmation of these inner heliospheric density values relative to pulsar dispersion measures are described in detail.

The 3D reconstruction technique begins with constant values of density and radial speed, and a daily cadence source surface inner boundary that we usually set at 15 solar radii. These values are projected outward by the UCSD kinematic model. LoS segment 3D weightings and the corrections to the density and velocity values from those observed are projected back in space and time, using a programming algorithm we term a “traceback matrix”, to the inner boundary source surfaces. The traceback matrix gives the origin location and time of each solar-wind volume element on the source surfaces. This matrix also provides the differences (in density and velocity) of the material in the volume element from those of its origin. The inner-boundary values are formally inverted to minimize modeled and observed differences between IPS $g$-level (modeled from the solar-wind density) and provided as new values of velocity and density on these surfaces. These inner-boundary Carrington maps of velocity and density are smoothed using spatial and temporal filters. The values are then again projected outward by the kinematic model for the next iteration. Iterated 9 times, IPS values discrepant by more than 3 sigma are removed, and the boundary values are again iterated. Final values are insensitive to starting source surface values and most of the convergence occurs within the first one or two iterations (Jackson et al., 1998, 2008a, 2008b).

Incorporating the magnetic field into this analysis is crucial for a full understanding of the physics of the heliospheric solar wind as well as Solar Energetic Particle (SEP) transport. For many space-weather effects, especially at Earth, the magnetic-field directions of the transient heliospheric structures are important in determining how interactions with the magnetic fields proceed. The Current Sheet Source Surface (CSSS) magnetic-field model (Zhao and Hoeksema, 1995) is used as an extension to the UCSD time-dependent analysis (Dunn et al., 2005). The CSSS model provides an accurate radial magnetic field, which is now updated every six hours at the inner-source surface of the tomographic analysis using National Solar Observatory (NSO) Global Oscillation Network Group (GONG) data (gong.nso.edu/data/magmap/). We extrapolate this radial magnetic field upward (in a forward model) to locations within the 3D matrix. These fields are primarily those of the background solar magnetic-field structure and do not include either the normal component of the radial, tangential, normal (RTN) fields relative to the Sun’s rotation, or the most dominant fields present from the field current that expels the CME. However, analyses covering a ten-year period of GONG magnetogram extrapolations show that background solar-wind magnetic fields are well represented in this way (see Jackson et al., 2016). In Jackson et al. (2019) these component-field analyses converted to geocentric solar magnetospheric (GSM) coordinates show that the Bz field component provides an accurate few-day forecast of substorm activity, primarily around the time of the spring and fall of the year.

## The 10 March 2022 IPS CME Forecast

The primary forecast in the past has always used and compared the 3D reconstructed plasma parameters forecast with Advanced Composition Explorer (ACE: McComas et al., 1998) density, velocity, and magnetic-field components. This is because these in-situ data have generally been available from NOAA within a few minutes of real time. Comparisons with the IPS data sets use one-hour averages that are smoothed to compare with the low resolution (6-hour cadence) of the 3D reconstructions. In more recent years exploration of other data sets for this use in density analyses from the Solar and Heliospheric Observatory (SOHO) Charge, Element, and Isotope Analysis (CELIAS) instrumentation (Hovestadt et al., 1995) has been used, since the data latency from this instrument is generally an adequate up-to-date reference for the UCSD 6-hour forecast analyses. Even more recently, and with ACE data becoming less continuous, we have explored using other in-situ near-Earth data sets in our forecast analyses, including from the Wind spacecraft (Ogilvie and Desch, 1997), and the Deep Space Climate Observatory (DSCOVR) instrumentation (Loto’aniu et al., 2022); see the UCSD website at ips.ucsd.edu/experimentalforecasts. In analyses of archival data sets the STEREO A and STEREO B Plasma and Suprathermal Ion Composition (PLASTIC: Galvin et al., 2008) measurements have also been compared to the IPS 3D reconstructions (Jackson et al., 2011b), Ulysses (Fujiki et al., 2003; Kojima et al., 2007; Hayashi, Tokumaru, and Fujiki, 2016; Tokumaru et al., 2021), and data from the Mars Global Surveyor mission (Jackson et al., 2007).

**Figure 3:**
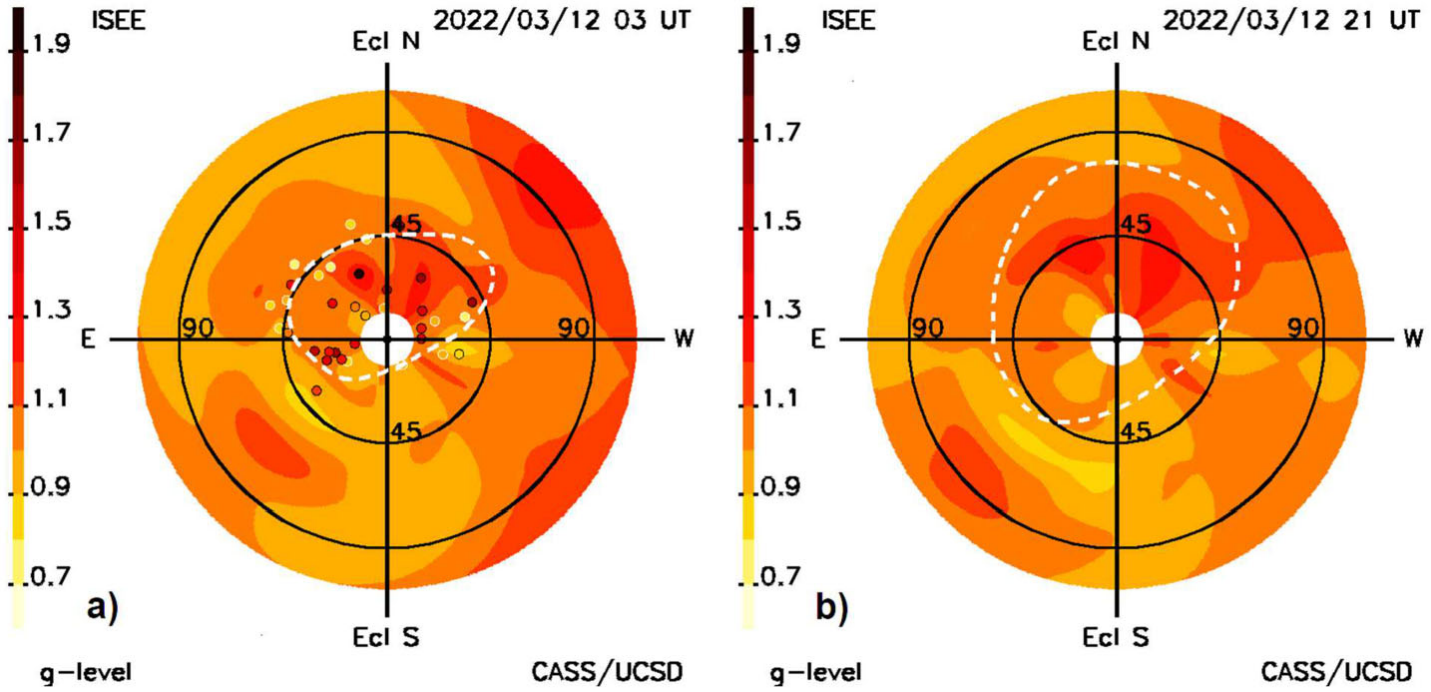
**a**) The 10 March 2022 halo CME as observed at 03 UT on 12 March in an IPS $g$-level “fisheye” sky map with the Sun at the center of the image that extends outward to 110° elongation; the inner circle near the Sun gives the 11.5° location below which strong scattering impedes $g$-level radio observations at 327 MHz. Also shown as small circles are the LoS positions of the radio sources present within ± three hours of the time indicated. The dashed line gives the approximate location of the outer extent of the halo CME at this time as measured in the density maps. **b**) The IPS $g$-level fisheye sky map is repeated from Figure 1b showing the outward expansion of the halo CME.

**Figure 4:**
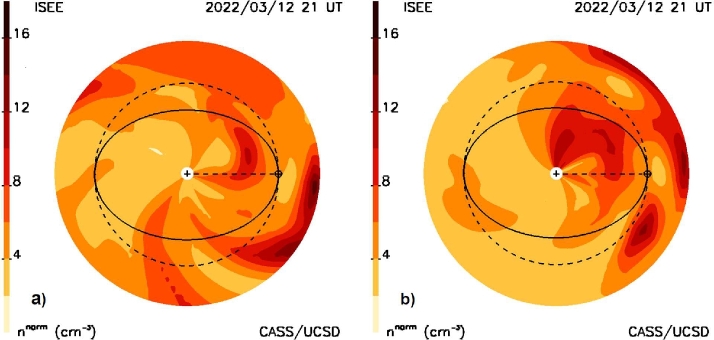
**a**) The 10 March 2022 halo CME as a fitted density model cut is shown rotated around the dashed Sun–Earth line counterclockwise as seen from Earth as viewed by the observer from 45° south of the ecliptic plane. Earth ($\boldsymbol{\oplus}$) is situated on its orbit to the left that is projected as an ellipse onto this cut through the volume. The dashed circle indicates the location of the Earth’s distance. **b**) A density cut rotated clockwise around the Sun–Earth line as seen from Earth and viewed from 45° north of the ecliptic plane.

**Figure 5:**
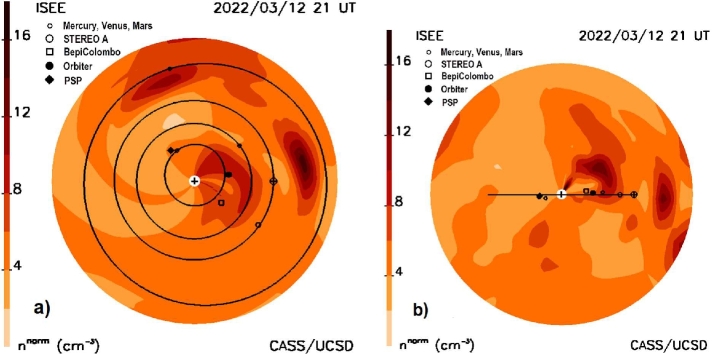
**a**) The 10 March 2022 halo CME as a fitted density model ecliptic cut is shown at the same time as in Figure 1c. The Earth ($\boldsymbol{\oplus}$) is situated on its orbit to the left. The inner planets to Mars, their orbits, and the spacecraft Parker, BepiColombo, Orbiter, and STEREO A are located projected onto the ecliptic plane. **b**) A density meridional cut at Earth is shown with all the planets and inner spacecraft projected to this plane.

**Figure 6:**
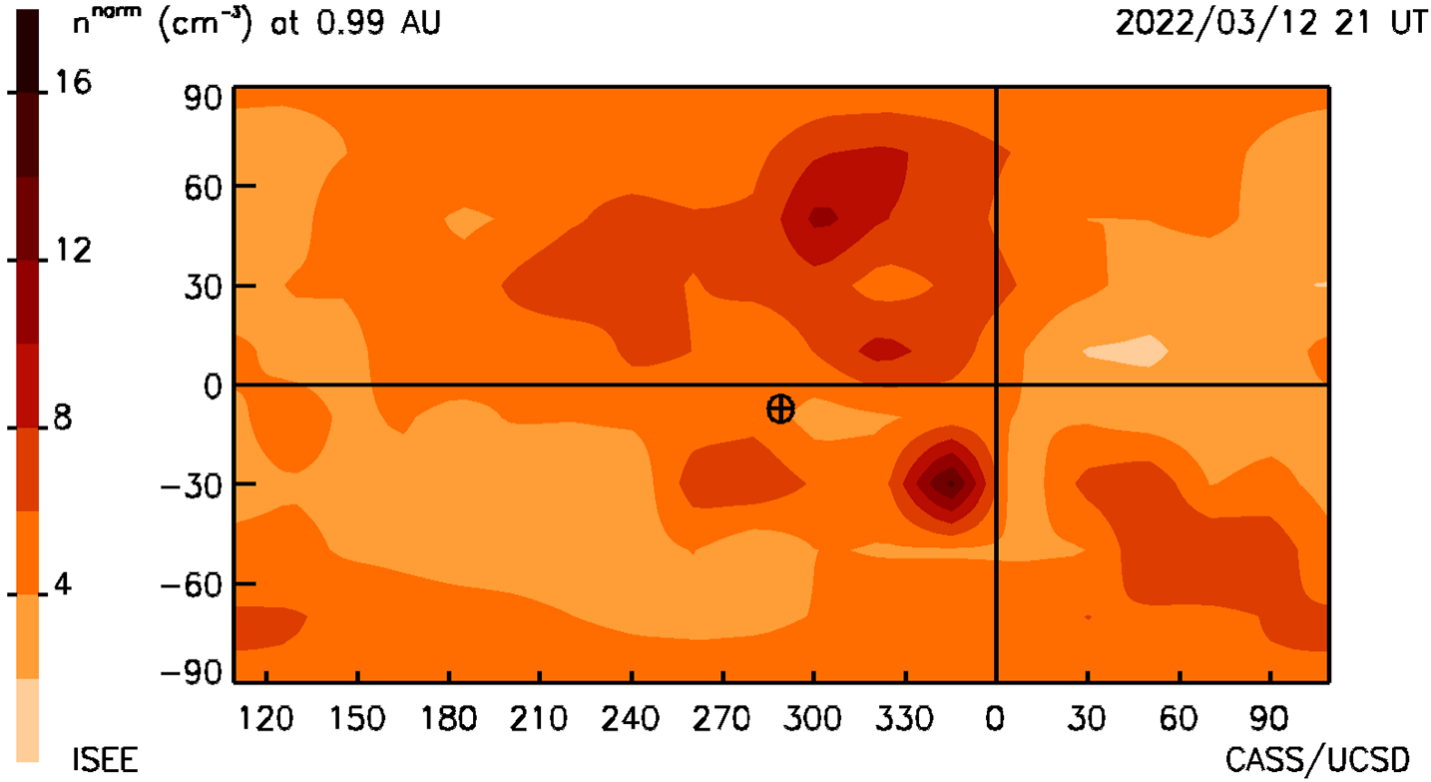
A heliographic synoptic map at the distance of Earth at the time of Figures 3b and 4. Earth ($\boldsymbol{\oplus}$) is situated near the center of the map. The structure to the north of the Earth is part of the 10 March CME that has arrived at 1 AU earlier than the material directed towards Earth.

**Figure 7:**
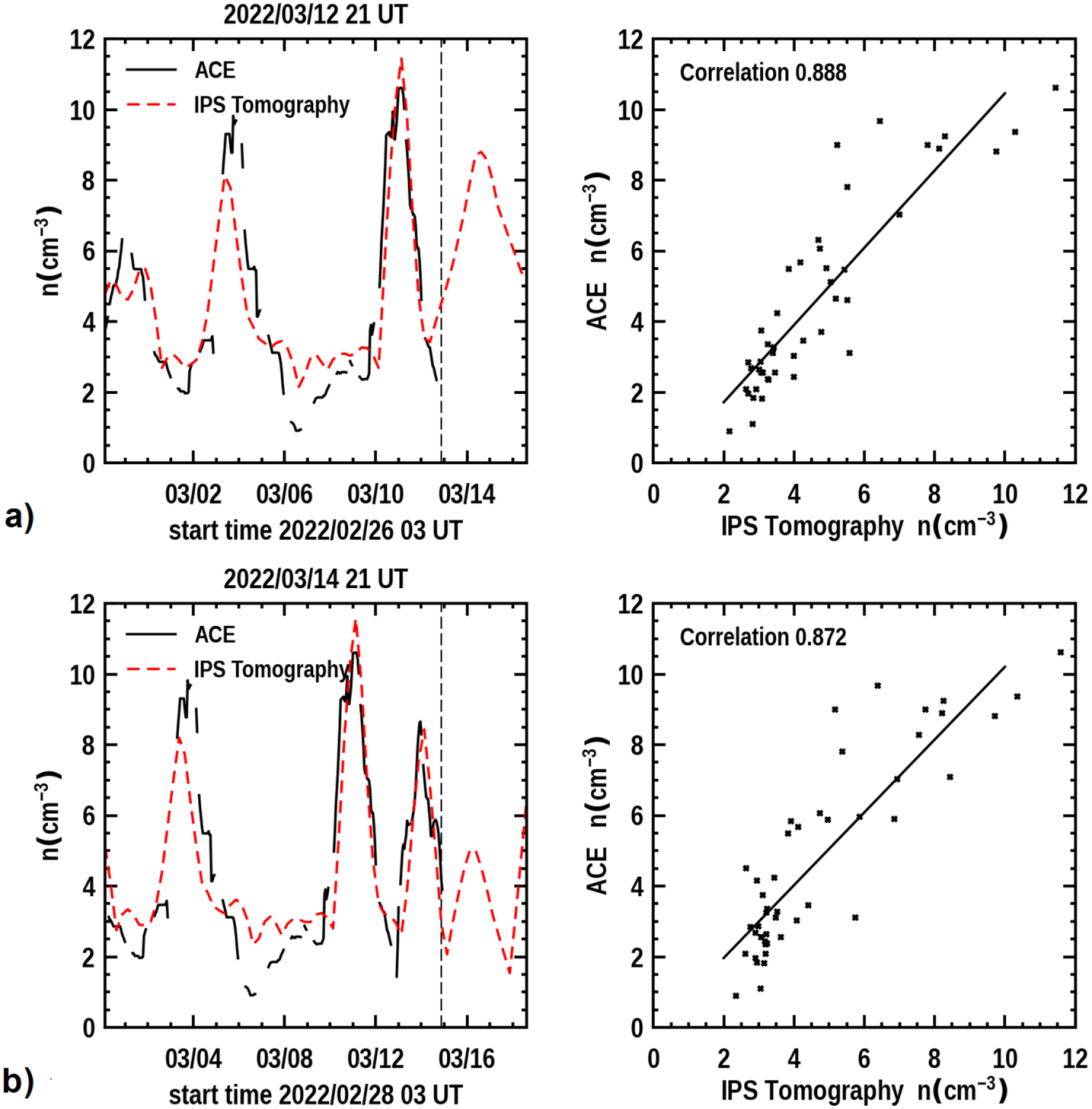
**a**) The 3D reconstructed density time series at Earth (dashed line) is shown to the left and compared with ACE in-situ density data for the same period that was available up to 17 UT 12 March 2022 at the program run time. The time at the top of the plot indicates the program forecast time at 21 UT, and this is given as a dashed vertical line in the image. To the right is a Pearson’s “R” correlation provided for the 6-hour cadence analyses (where data are available from ACE). **b**) The 3D forecast reconstructions for the ISEE analyses normalized with and compared to ACE data on 21 UT 14 March 2022.

**Figure 8:**
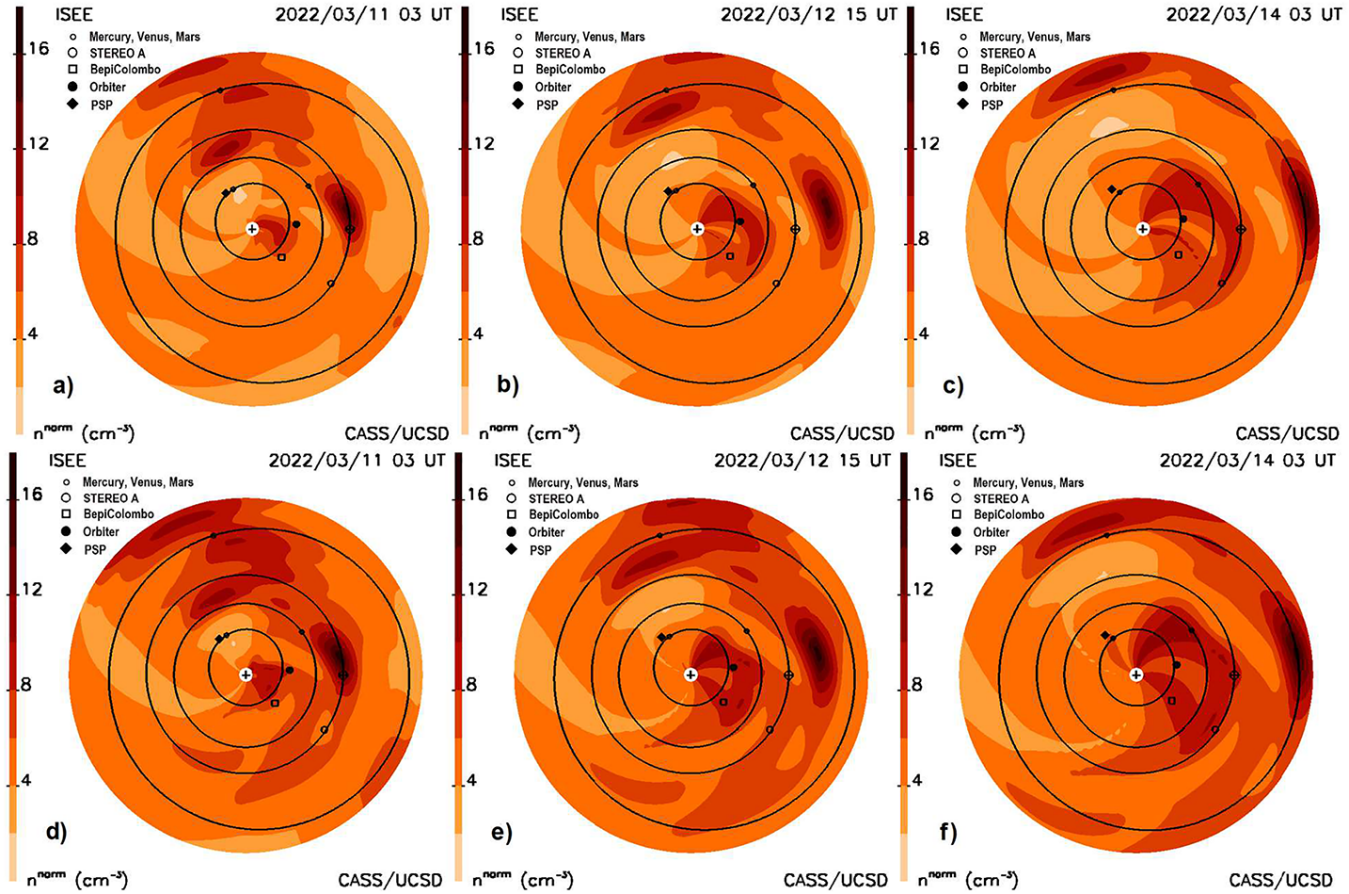
**a–c**) The 10 March 2022 halo CME density is shown in this sequence of ecliptic cuts normalized to ACE in-situ data. **d–f**) Normalized to Wind data using the same IPS data set. The two ecliptic cut sequences are very similar, showing that the CME has reached BepiColombo and Solar Orbiter at the beginning of the sequence, and at the end of the sequence shows that the CME has fully arrived at Earth.

Figure 1 shows the forecast of the CME as it was presented on the UCSD website on March 12, at 21 UT. Additional forecast analyses available at the time of the CME are shown in Figures 3 – 8. In Figure 3 the outward expansion of the halo CME is shown as a fisheye skymap. Figure 3a also shows the LoS positions of the ISEE radio sources within three hours of 3 UT that were used to fit to the model for the full day of ISEE data prior to the 21 UT IPS forecast. These are only the small immediate portions of the fit to 1536 sources, since all the $g$-level sources from previous days plus those until the last source (0422+178) was observed on 7:58 UT March 12 were used in the spatial and temporal analyses. These radio sources are coded with their actual scintillation level and this is circumscribed in black if the source value is greater than the value of the model at the time they are compared, or conversely circumscribed in white for less value. From that time onward, the LoS fits to the model are extrapolated outward so that $g$-levels, densities, and other remotely sensed solar-wind parameters are forecast values. Only when additional data become available can a new analysis be presented by the UCSD time-dependent program. ISEE IPS data are edited throughout the night following their being obtained, and then on the next day these data are placed on their website, nominally at ∼17 UT. Following a UCSD query that these data are available at ISEE, they are then downloaded, and the 3D reconstruction analysis program is begun. The reconstruction takes less than an hour to complete and provides the images shown in the forecast analyses for a 20-day period at a six-hour cadence. Thus, the data latency from when ISEE data are obtained near the Sun at 3 UT until they are available to be viewed, is about 15 hours, or at ∼18 UT. From the time the last source of the day is observed the latency is about 10 hours. These analyses are then shown at the next six-hour interval centered on the approximate noon-time value at ISEE, or at 21 UT. The other 3D cadence reconstructions from 17 UT onward usually proceed at a six-hour run-time cadence, are normalized by in-situ measurements up to this time; these are projected outward and compared with the real time in-situ data available up to the reconstruction run time. Figure 3 also shows a structure with high $g$-levels seen to the solar northwest. This is a portion of a CME that erupted from the Sun to the solar northwest early on 8 March 2022. The structure to the solar southeast that appears between 45° and 90° in Figure 3a and in Figure 3b at 90° is a portion of another CME that erupted on March 9.

Figure 4 provides retrospective analyses for these volumetric data; it shows a sample of two cuts for this event that have been rotated 45° counterclockwise and clockwise viewed from Earth relative to the Sun–Earth line. In addition to the ecliptic and Earth meridional cuts these show the approximate propagation location of the portions of the CME or CME group on 21 UT March 12 on the way outward from the Sun. In Figure 4, and other density cuts shown in this article, an r^−2^ fall-off has been removed from the volumetric data to provide a more consistent representation of heliospheric structures as they move outward. To determine the CME’s outer extent, the location of the event at the contour level 6 n^norm^ was used as a marker above the ambient solar-wind value that is generally ∼5 Np at Earth. In Figure 4a the observer is looking at this plane from the east of Earth from 45° *below* the ecliptic. Earth’s ecliptic orbit is projected onto this plane as an ellipse. The dashed circle indicates the 1 AU distance from the Sun; this shows that a portion of the CME has probably reached 1 AU at this time to the northeast of the Sun–Earth line. Similarly, Figure 4b provides a meridional cut relative to the Sun–Earth line as viewed by the observer positioned 45° *above* the ecliptic. From this position we see that the CME has indeed reached 1 AU at this time, but has not yet done so in the ecliptic. Similar cuts at 3 UT as well as the ecliptic and Earth meridional cuts of Figure 1 are used to determine the approximate location of the outer extent of the CME shown as the dashed lines in Figure 3. It is clear from the forecast volumetric data sets that the outward-moving material seen in the sky maps does not necessarily match very well with the outer edge of the CME seen in the various data cuts. However, some of the main indicators of the oncoming CME are the sources near the Sun in Figure 3a that have very high levels of scintillation relative to the model values at that time.

From the same volumetric data set as Figures 1, 3, and 4, Figure 5 provides ecliptic cuts and an Earth meridional cut of the 10 March 2022 CME as it was forecast on the UCSD website at 21 UT showing orbits and positions of the inner planets. Figure 5a also shows the heliospheric spacecraft Parker, BepiColombo, Orbiter, and STEREO A as projected onto the ecliptic plane. Figure 5b is a meridional cut at Earth with the inner planets and spacecraft projected to this plane. Given that the CME was observed as a halo, and that each cut shows that the structure is observed east–west and north–south of the Sun–Earth line, the CME is certain to reach Earth. Solar Orbiter is nearly on the Sun–Earth line, while BepiColombo is aligned with STEREO A, but shown in the meridional cut to lie above the ecliptic plane and in the void area behind the dense portion of the CME at the 21 UT time depicted in the 3D reconstruction. Figure 6 is a synoptic cut at Earth’s distance (0.99AU) at 21 UT March 12; here the outer extent of the 10 March CME is present north of the Sun–Earth line, especially to the northwest, as has also been indicated in Figure 4.

Figure 7 provides a time series of the density and the 3D reconstructed velocity near Earth showing earlier structures over 14 days that have passed the ACE spacecraft situated near the first Lagrange point of Earth (L_1_) as well as a forecast of the density expected to arrive at the ACE for four days following. The vertical dashed line is the last UT time that the 3D reconstruction program was run. This low-resolution analysis indicates the arrival of the 10 March CME is imminent. With the knowledge that the 3D reconstructions are provided with a one-day cadence and interpolated at 6-hour intervals, this indicates that the CME should fully be present at Earth within about 12 hours. The in-situ time-series data is smoothed with a one-day boxcar filter to have approximately the same amplitude variation as the low-resolution 3D reconstruction analysis. It also shows that the CME follows shortly after a portion of what appears to be the 8 March CME that was also listed as a prior halo event. That the 10 March CME did arrive at Earth with the approximate timing and amplitude of the expected density enhancement is shown in Figure 7b in a later forecast on 21 UT on March 14, 2022 two days following the analysis shown on March 12.

## Additional 3D Reconstruction Analyses of the 10 March 2022 CME

### 3D Reconstruction Forecast Consistency

**Figure 9:**
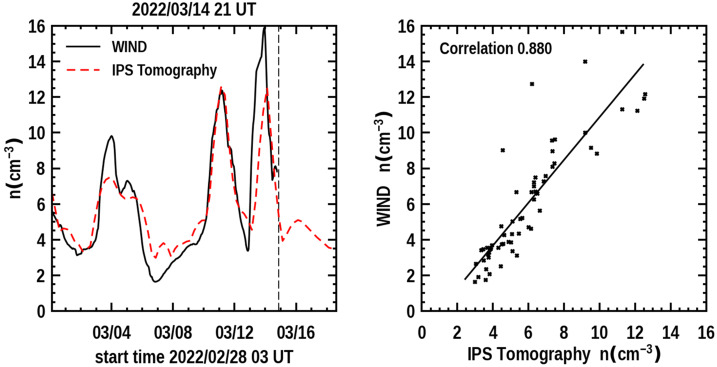
The 3D forecast reconstructions for the ISEE analyses normalized with and compared to Wind data on 21 UT 14 March 2022. This analysis can be compared with Figure 7b, but note the slight ordinate-scale difference.

That these forecasts yield consistent results near Earth can be demonstrated in many different ways. The least-squares fits become increasingly smaller throughout the iterations. Figure 3a shows the least-squares fits of the modeled LoS analyses compared to the observed LoS, but for the instantaneous values obtained over the approximate three-minute interval for each observation. Furthermore, the least-squares fits on the source surface where the differences between measured values and observed values are compared also become much less throughout the iterations (see Jackson et al., 2008a). The other ways are through comparisons of the in-situ measurements to the modeled values, as in Figure 7, or by comparison of the future volumetric data that also fit retrospectively to the in-situ values as in Jackson et al. (2010, 2013). Other tests can be made as in Figures 8a–c that present a forecast sequence of the ecliptic cuts present at 21 UT March 12, 2022, from the IPS analysis. Here, this CME is shown to progress outward over the period from 03 UT March 11 until 03 UT March 14 at a 1.5-day cadence; Figure 5a is from three hours later than the second image in this sequence. The IPS and in-situ data for these analyses are the same for those shown in the figures through to Figure 7a in the previous section. In Figures 8d–f the same IPS data are shown normalized with Wind in-situ data up until the computer run time at 17 UT March 12. These forecast analyses are fairly similar even though the in-situ data have a slightly higher overall amplitude (see Figure 9). The analyses show that the CME has just reached BepiColombo and Solar Orbiter at the time of the first image, and also that an eastern portion of the May 10 CME has arrived at Earth. By the time of the last image in the sequence, the CME has fully engulfed Earth. The in-situ analyses for this period can be compared with Wind data shown in Figure 9 that comes from a forecast at 17 UT on March 14 as in Figure 7b where more IPS and in-situ data are available. In comparison with earlier in-situ forecast analyses from March 12, the CME is more refined and decreases in density following its initiation. The analysis in this instance is shown to follow the smoothed ACE data better than it does Wind for this event.

**Figure 10:**
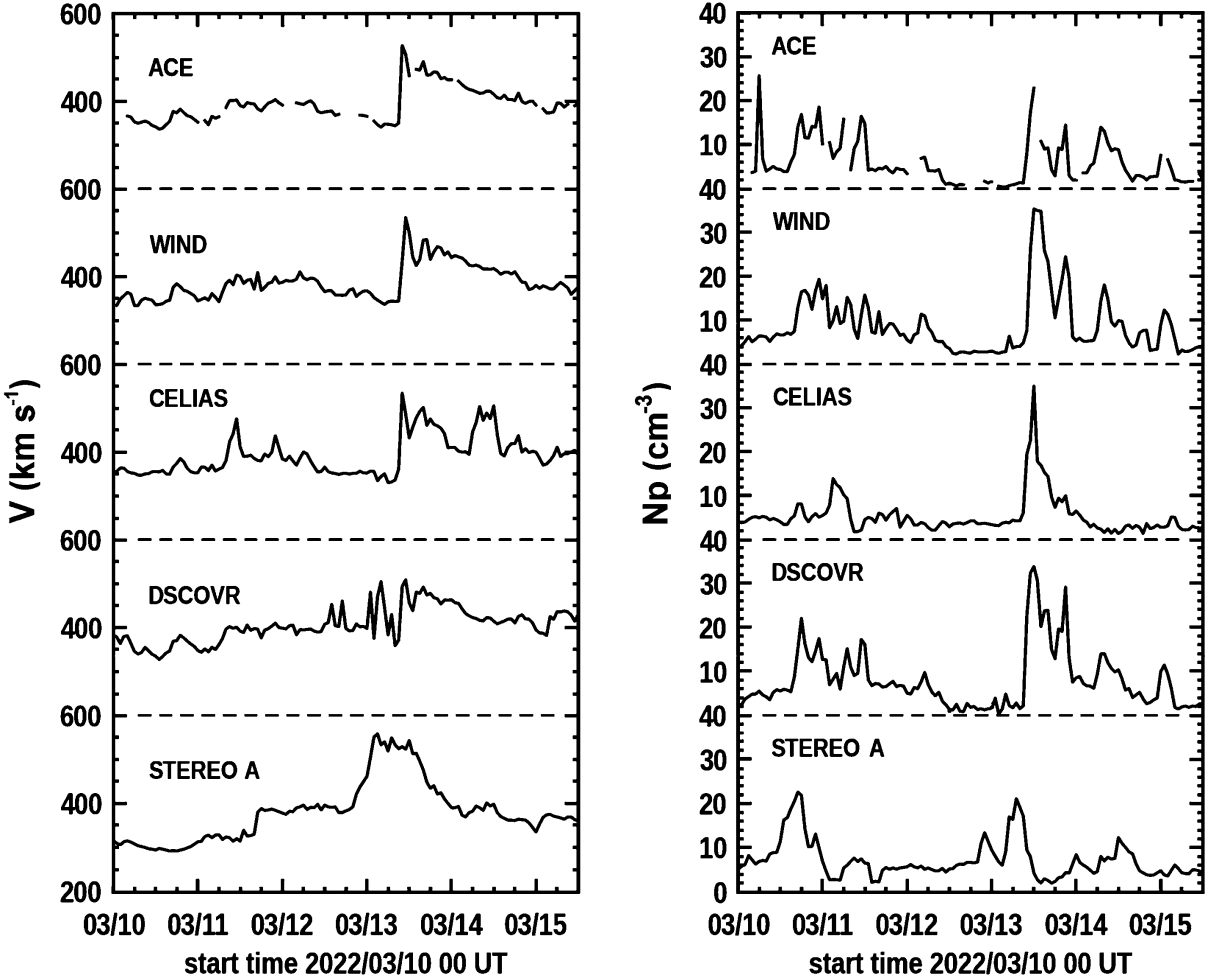
In-situ one-hour averaged measurements of velocity and density for the five spacecraft at 1 AU from the period of 0:00 UT 10 March to 12 UT 15 March 2022.

Figure 10 shows one-hour averages of velocities and densities from all four near-Earth spacecraft ACE, Wind, CELIAS, and DSCOVR, and for STEREO A situated on the ecliptic 34° east of the Sun–Earth line during the interval from 10 March 0 UT through 15 March 12 UT. The density hour averages are considerably different during this period from the different spacecraft near Earth. In these comparisons we note that the Wind, CELIAS, and DSCOVR data are more complete than from plasma velocity and density from ACE for this period, but that for the 10 March and earlier 8 March events much of the solar-wind density amplitude is present in small-time-scale short-lived structures whose amplitude cannot be accurately reproduced in the UCSD 3D reconstruction analysis. There is a dominant shock-fronted structure that provides much of the enhanced plasma especially when the 10 March CME arrives near Earth with an onset at 11:00 UT March 13. We note that the CELIAS density data shows well the onset of the 10 March CME, but is otherwise considerably different from the other three instruments near Earth. The DSCOVR velocity data at this time show the 10 March CME shock onset, but has a great deal more variation and a higher average velocity prior to the event onset. While some of the discrepancy is expected due to the different instrument locations near L_1_, we presume that most of it results from the different systems each instrument uses to monitor solar-wind plasma.

In further explanation of the matter of different measurements from the four L_1_ spacecraft, we note that many of the hourly average excursion differences are greater than a factor of two. The distances between L_1_ spacecraft generally average about 10 – 30° from one another observed from Earth. At the times shown in Figure 10, the distance of Wind from SOHO had the largest separation of 32^∘^ as seen from Earth; the other three L_1_ spacecraft were clustered within ∼10° of one another. At the average speed of 400 km s^−1^ the solar wind passing an L_1_ spacecraft would travel ${\sim} 1.5\times 10^{6}\text{ km}$, or the distance of the L_1_ point to Earth. To provide a factor of two excursion difference, two radial columns the distances of the spacecraft would need to maintain a factor of two difference over their entire length that, for all but Wind, would have had to been over five times greater in the solar radial direction than the distance of each spacecraft from one another. By this same line of reasoning, a CME shock front passing these same spacecraft would need to be inclined or measured in arrival by 80° relative to the tangential (or GSE Y) solar-wind direction to provide a timing difference that is more than an hour. This implies a radially structured heliosphere that does not randomly average away in the course of an hour. While mesoscale solar-wind structures can be imagined at 1 AU solar distances, these are generally not expected or measured to be organized into radial-density inhomogeneities (Borovsky, 2012; Lugaz et al., 2018). Thus, to summarize, we expect for hour average excursions, the spacecraft location differences are small relative to the one-hour distance travel times of the solar wind past each spacecraft; and thus it is likely that the hourly differences in density measurement are mostly instrumental.

### Forecast Comparisons with Other Interplanetary Spacecraft

**Figure 11:**
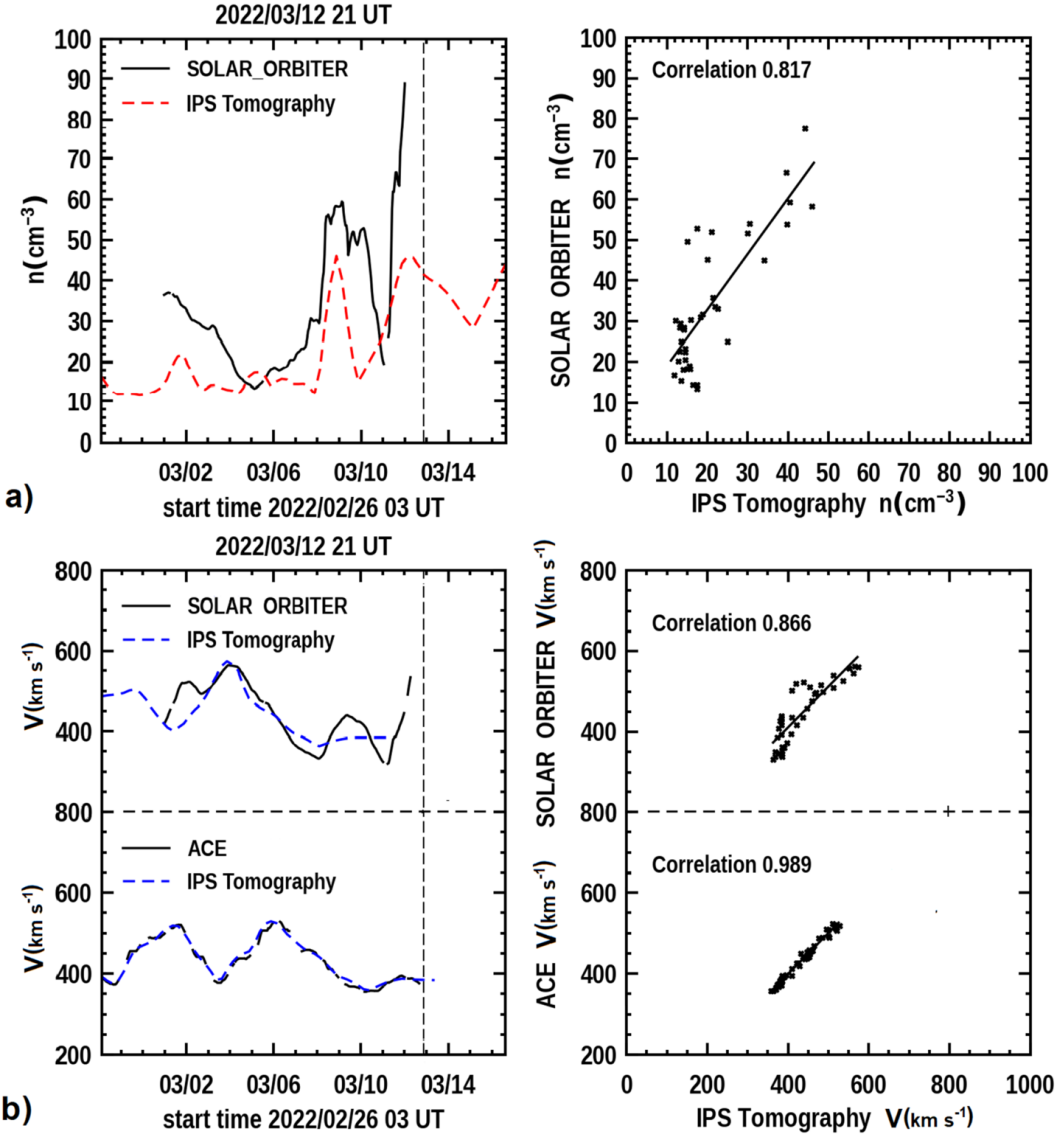
**a**) The 3D forecast reconstructions for the ISEE analyses normalized with ACE data and compared to the Solar Orbiter plasma density data as forecast on 21 UT 12 March 2022. **b**) 3D reconstructed velocity compared with in-situ measurements for both Solar Orbiter and the ACE spacecraft.

The 10 March 2022 CME had a good radial alignment between two inner planetary spacecraft and others at 1 AU, and in this way details of the outward propagation of the CME can be checked. Solar Orbiter was about 10° west of the Sun–Earth radial and 2.8° north of the ecliptic at the time of the CME arrival. Following the CME eruption at the Sun, the magnetic-field data for this event arrival was measured at Solar Orbiter prior to the CME Earth arrival (Palmerio, 2022), at ∼19:50 UT March 11, and was predicted to reach Earth by Möstl (2022) at ∼6 UT March 13 that included a shock response midday on that same day. In fact, the CME was observed to arrive near Earth with a shock response at 10:09 UT determined by the shockspotter system (Kruparova et al., 2013) in the CELIAS in-situ measurements. Figure 11a shows the UCSD 3D density reconstruction result and its comparison with Solar Orbiter density data. The analysis has a fairly good low-resolution correlation.

Again, as for at Earth, the in-situ data that have been averaged with a one-day box-car smoothing function does not show the large shock responses present in the in-situ analyses. Additionally, the average total response is considerably lower in total overall density. At this time of year there is no velocity data from the IPS system in Japan due to the closure of the two movable mountain antennas that cannot function when snow loading occurs. As a substitute for this, we regularly insert in-situ velocity from the near-Earth spacecraft into the 3D reconstruction analysis (see Jackson et al., 2010). This provides a match of the tomographic analysis at Earth and throughout the near-Earth volume at the resolution of the volumetric data as shown on the lower panel of Figure 11b. The 3D reconstructed velocity that occurs prior to that Earth distance is shown in the upper panel of Figure 11b, and this matches well the measured Solar Orbiter in-situ velocity, but is not capable of providing a forecast into the future.

**Figure 12:**
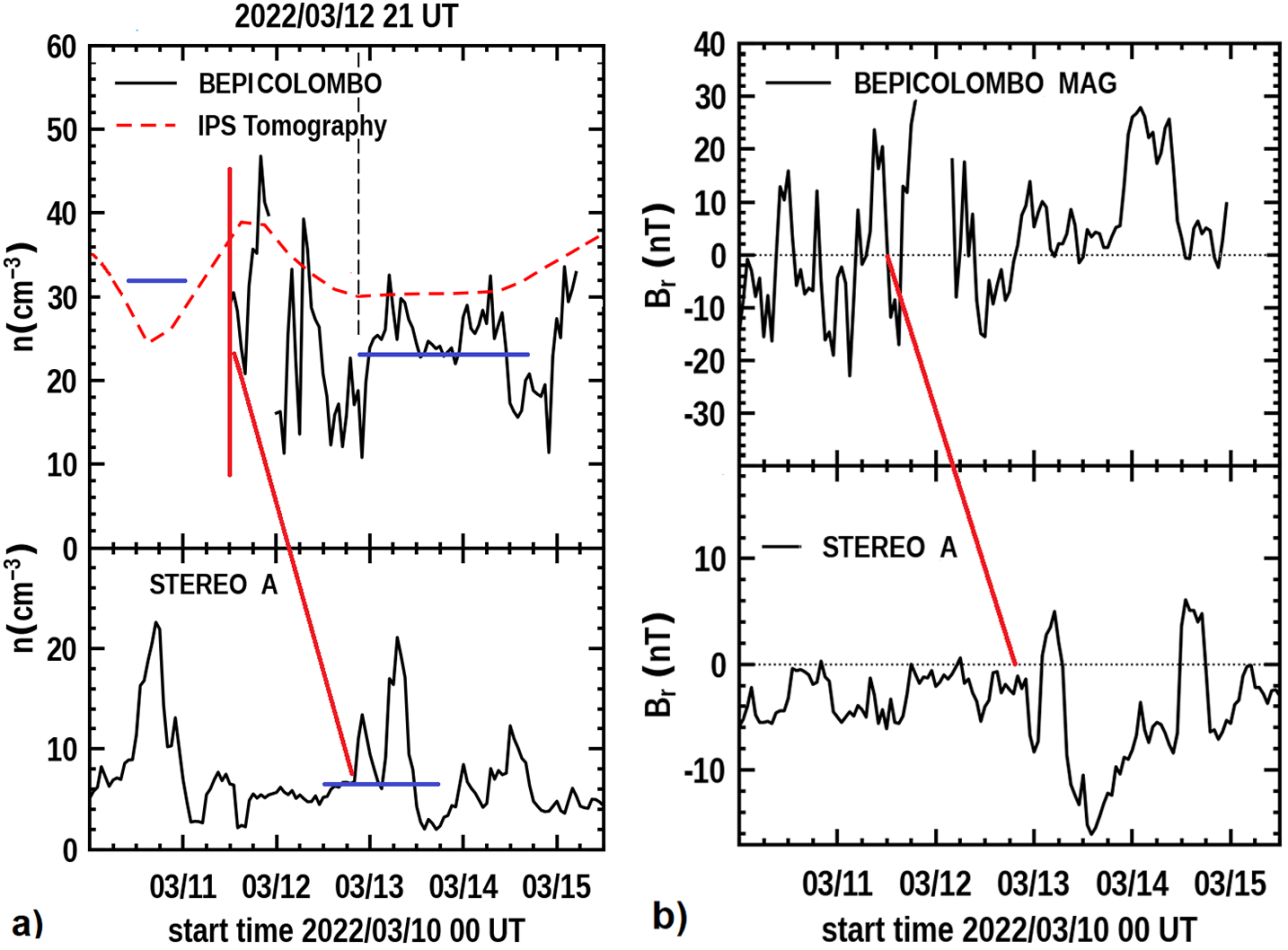
**a**) (Top Panel) The 3D density reconstructions as forecast on 21 UT 12 March 2022 from the ISEE analyses normalized with ACE data is compared to the BepiColombo MEA electron density data that have had the spacecraft potential field approximated. (Bottom Panel) Measured STEREO A in-situ proton density replotted from Figure 10. The vertical red line indicates the onset time of the Forbush decrease observed in the Mio SPM instrument. The diagonal red line marks the approximate offset of the CME onset in the two time series. **b**) (Top Panel) BepiColombo hour-averaged radial field over the same interval as in Figure 12a. (Bottom Panel) STEREO A hour-averaged radial field. The ends of the diagonal line indicate the CME onset times as in Figure 12a.

At the time of the CME event arrival, BepiColombo was about 5° east of the Sun-STEREO A radial that was 34° east of Earth, and 6.8° north of the STEREO A ecliptic latitude. The analyses from the 3D reconstructions and BepiColombo and the STEREO A in-situ measurements during this period are shown in Figure 12a. The bottom panel of this figure provides one-hour averages of STEREO A PLASTIC science data throughout this period. The top panel of Figure 12a gives the 3D reconstructed density forecast as normalized by ACE, and the one-hour averaged in-situ density measurements at BepiColombo. The BepiColombo plasma electron densities are measured by the Mercury Electron Analyzer 1 (MEA1) of the Mercury Plasma Particle Experiment (MPPE) instrument suite (Saito et al., 2021) onboard the JAXA-led Mio spacecraft of the BepiColombo mission (Murakami et al., 2020). We note that the BepiColombo electron densities shown here are best estimates assuming the electron plasma is isotropic and relying on 3D data products where only the two anodes of MEA1 unobscured by the Magnetospheric Orbiter Sunshield and Interface Structure (MOSIF). MOSIF provides thermal protection and the mechanical and electrical interfaces for Mio during the BepiColombo cruise phase. In the absence of a direct determination of the spacecraft potential by the Wire Probe antenna from the Plasma Wave Investigation (PWI: Kasaba et al., 2020), stowed during cruise phase, the floating spacecraft potential is estimated following a procedure described by Lewis et al. (2008). Although all the limitations raised above can still cause large unknown density variations from MEA measurements, the best estimates of MEA1 electron densities during the second BepiColombo Venus flyby (Persson et al., 2022) compared reasonably well with the proton densities derived at a distance of about 1.2 million km by the Solar Wind Analyzer (SWA) onboard Solar Orbiter (Owen et al., 2020).

Although we cannot reconstruct density at the in-situ cadence, both time series show an enhancement on 11 March at the time the UCSD 3D in-situ reconstruction or the ecliptic cuts of Figure 8 show the CME arrival at BepiColombo. Generally, a Forbush decrease beginning can be directly linked to the CME sheath magnetic-boundary onset (Davies, Winslow, and Lawrence, 2023). The MEA1 data are not available from before the CME is present, and as an additional way to time the event onset we have marked the initiation of the event in Figure 12a by a vertical line at the beginning of a Forbush decrease observed by the Solar Particle Monitor (SPM1 and SPM2) from BepiColombo at 12 UT 11 March. This is also consistent with the onset time of the CME marked by the ESA MAG experiment on BepiColombo shown in hour averages of the radial field in Figure 12b. The STEREO A IMPACT (Luhmann et al., 2008) measured radial field also shown in Figure 12b gives the same indication of the 10 March CME onset as do the STEREO A proton measurements.

As is shown in Figure 12a, the in-situ electron densities from the BepiColombo MEA1 measurements and the UCSD reconstruction analyses are in relatively good agreement. The approximate amplitude difference between the two time series shown by the horizontal blue lines indicates the response given by the forecast is about 1.38 times that measured by MEA during this period. Another small factor must be included for this difference since the IPS analysis (that is determined from electron variations) has been normalized to the proton number to compare more readily with in-situ densities (see Tiburzi et al., 2022). If we assume a 4% average solar-wind helium number abundance (Kasper et al., 2007) this adds another 8% electron number to this factor, providing a MEA instrument normalization factor of 1.5 for this time period. When the potential field factor is not subtracted from the time series, and a Maxwellian peak, density determination made, the time series is equal to or somewhat higher over the whole of the March 2022 interval. However, for these measurement values, the excursions relative to those observed in the 3D reconstructed analyses are much greater over this interval, and do not agree as well with few-day peaks and valleys present in the reconstructions. The onset of the CME from the Forbush decrease and the BepiColombo MEA analysis is present 42 hours prior to the double-peaked enhancement of the 10 March 2022 CME arrival at STEREO A and occurs at about 18 UT 12 March, 17 hours before the CME arrives at Earth.

The STEREO A one-hour averaged data are presented in the bottom panel of Figure 12 for comparison with the BepiColombo data, and the red diagonal line marks the delay of the CME reaching STEREO. We can further certify that these two structures are the CME and that the analysis presented as a factor of 1.5 is correct by using a few simple calculations. There is no value of CME velocity given at BepiColombo, but there is at STEREO A, and this shows a peak in-situ value speed of ∼500 km s^−1^ (Figure 10). Assuming the speed has remained constant from the 0.43 AU distance of BepiColombo to the 0.97 AU distance of STEREO A (a difference of 0.54 AU), this provides a radial difference travel time between the two spacecraft of 41 hours or a transit time speed value very close to the speed actually observed at STEREO, and thus this is the CME. This also implies that there has been little change in the outward motion speed of the CME from BepiColombo to STEREO. The value of density from the 3D reconstruction is about 32 Np. Since over this distance the CME has a constant speed and probably little expansion other than radial outward flow, the decrease in the solar-wind density to conserve mass flux has a value of $32\times (0.43/0.97)^{2} = 6.2\text{ Np}$, and this is marked by the blue horizontal line superimposed on the double-peaked enhancement on the STEREO A in-situ time series. Thus, the 3D reconstructed density at BepiColombo also fits the STEREO value, and the factor of 1.5 difference between the MEA and 3D reconstructed value is also shown to be valid from this standpoint.

**Figure 13:**
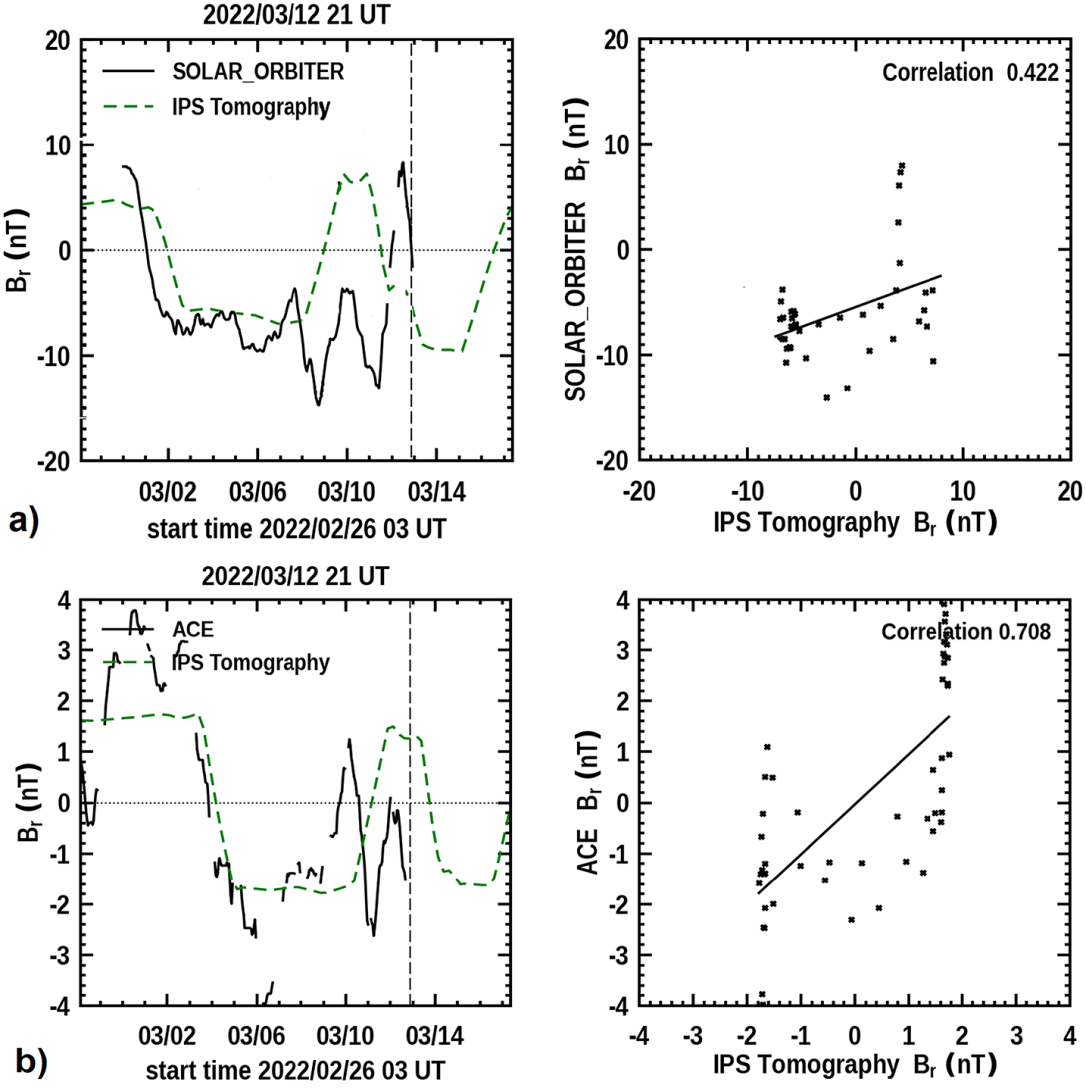
**a**) The 3D radial magnetic-field component reconstruction in RTN coordinates from the CSSS model as presented on 21 UT March 12, 2022 compared with the measured Solar Orbiter radial field. **b**) 3D reconstructed field compared with the measured radial field from ACE.

We provide a forecast of magnetic fields in our analyses to show how well the component fields of our CSSS model fit these fields near Earth both as RTN fields and in GSM Bz (Jackson et al., 2019) at ips.ucsd.edu/high_resolution_predictions. The modeled fields are extrapolated outward from the solar surface, and the GONG surface fields refreshed every six hours. Although these background solar-wind fields are shown to predict geomagnetic substorm activity, they do not compare well with rapid CME field variations except perhaps at CME onset times as reported by Nishimura et al. (2016). However, they are instructive in comparison with both the in-situ measured CME fields and attempts to forecast them. Figure 13 gives the modeled radial magnetic field, compared with both the ACE and Solar Orbiter measured radial field and the Pearson’s R correlations of these field comparisons. The low-resolution modeled radial field throughout the time period fits and gives a fairly high correlation with those measured, especially for the large-scale variations at the sector boundary crossing at Solar Orbiter and ACE on 2 March and 4 March 2022, respectively. The comparison breaks down significantly where the 8 March and 10 March CMEs pass their respective spacecraft. The component fields where these CMEs pass the Earth (ACE) are generally considered dominant in providing the large excursions relative to GSM Bz as the cause of geomagnetic storms but these generally only add to the background fields at these times.

**Figure 14:**
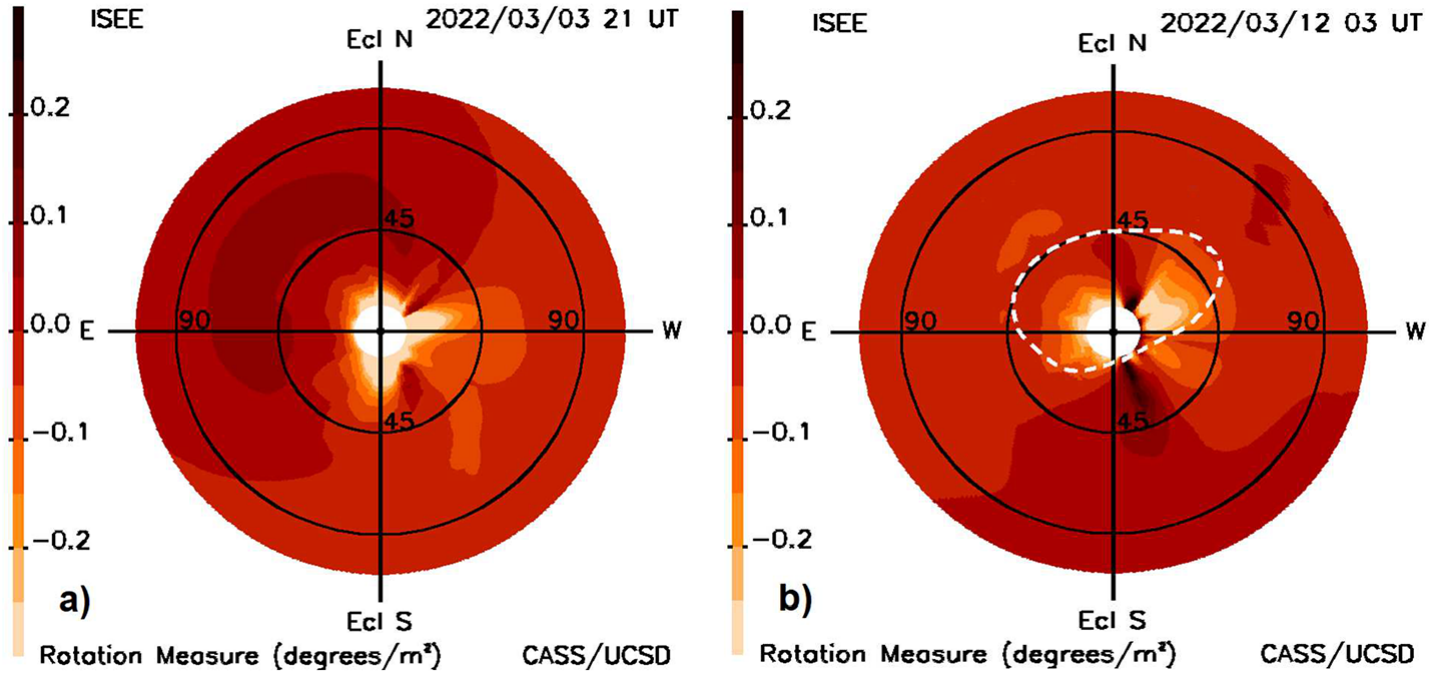
**a**) A “fisheye” rotation measure (RM) sky map with the Sun at the center of the image that extends outward to 110° elongation at 21 UT on March 3, 2022 at the time of magnetic-field change from positive to negative as observed from Earth. **b**) The RM forecast fisheye sky map at 03 UT on March 12, 2022. The halo CME contour from Figure 3a is superimposed on this map to locate the approximate position of the outer edge of the CME.

There has been considerable interest lately and experimentation and modeling using Faraday rotation to provide forecasts of magnetic fields (Jensen et al., 2010, 2023; Wexler et al., 2016; Kooi et al., 2021; Gopalswamy et al., 2023). Faraday rotation in the interplanetary medium is the linear integration of the LoS tangential field vector and density that gives the rotation ($\beta $) of the polarization amount of the electromagnetic field as it traverses along the LoS as given in Equation (1) as;

1 $$ \beta = \frac{e^{3} \lambda ^{2}}{8 \pi ^{2} \varepsilon _{0} m^{2} c^{2}} \int _{0}^{d} n_{e} \left ( s \right ) B_{\parallel} \left ( s \right ) \mathrm{d}s, $$

where $n_{e}$($s$) is the density of electrons at each point along the LoS, $B_{\parallel} $($s$) is the component of magnetic field in the direction parallel to the LoS, $e $ is an electron charge, $\lambda $ is the wavelength of the radiation, $\varepsilon _{0}$ is the vacuum permittivity, $m $ is an electron mass, and $c$ is the speed of light. In the case of heliospheric measurements at meter wavelengths from Earth to distant interstellar polarization sources, the top limit $d$ for the integral, for all practical purposes, is to the edge of the inner heliosphere since this is where the strongest large-scale fields and densities are present outside of the Earth’s ionosphere. Since different wavelengths of light can be measured at Earth, and all but the values beyond the integral sign are constant, a common way to describe Faraday rotation is by using Rotation Measure (RM), the value of $\beta $/$\lambda ^{2}$. If densities along the LoS are known, the LoS field strengths can also be determined. UCSD modeling regularly provides both two components of RTN fields (R and T) and densities in the interplanetary medium as 3D reconstructed volumetric data. Figure 14 shows RM fisheye sky maps similar to those of Figure 3, that UCSD regularly provides as forecasts derived from our global analysis of field component and density for two specific times during the time interval close to the 10 March CME transit period. In the presentations shown there is no fall-off applied to the images to show regions far from the Sun more clearly. These low-resolution analyses provide an integration of both the background fields and density from the CME and the background solar wind, and as such give a good indication of the RM values to be expected from heliospheric fields. That CMEs are present in these measurements is not very evident, mostly because of the large variations in the background solar-wind fields and densities that are also present along the LoS in the fisheye sky maps for this CME. Although higher resolutions may provide RM variations that detect sheath regions of CMEs or other locations of high electron densities and their associated fields, these analyses will also need to accommodate similar fields and densities present in the background solar wind.

## Better Forecasts and IPS Fits of the 10 March 2022 CME

The question always arises as to whether the IPS analysis could have provided a better forecast and the analysis could have been better fit, or whether different depictions might provide more information. For this event, perhaps cuts through the volumetric data other than those at the ecliptic or on the Earth meridian, would also give more information about the CME morphology.

In these analyses there is no such thing as a unique fit. Although the analyses converge to a smooth solution independent of starting parameters, the final fit is predicated by the amount of data present, and the amount of “noise”. This noise is present from both the IPS observations themselves and from approximations made in the model that fits the data along each LoS. Here, we have explored some of these potential differences as indicated by using Wind data to normalize our observations shown in Figure 8 and Figure 9. Indeed, normalization of the models using other than the ACE in-situ data has some effect as noted. For use in driving the ENLIL 3D magnetohydrodynamic (MHD) model (Odstrcil and Pizzo, 1999; Jackson et al., 2015, 2020) it has been suggested that the analysis provides a higher-cadence tomographic reconstruction and use six-hour cadence lower boundaries rather than those that are currently interpolated at six hours. Although this process is still ongoing, the current numbers of IPS LoS are sparse and thus far this exploration has been done at the expense of a better global analysis. In the case of the 10 March CME this higher cadence provides a less complete structure even though there is a better-resolved forecast cadence at Earth.

Other time-dependent IPS 3D reconstruction analysis techniques are underway, such as those using the ENLIL 3D MHD model rather than a kinematic model as a kernel in the IPS 3D reconstructions (Jackson et al., 2020). This is shown in real-time operation on the UCSD website ips.ucsd.edu/ENLIL_predictions. Another technique (SUSANOO-CME) uses many runs of MHD 3D simulations to fit IPS LoS (Iwai et al., 2019, 2021). The latter analysis requires assumptions to be made about the intrinsic nature of the CME such as its magnetic configuration (the shape of the Spheromak fit), its location, and other parameters specific to the CME relative to the background solar wind. Although these MHD analyses have the potential to determine the nonradial outward plasma-flow modification and magnetic configuration of a CME, these fields have so far not been well observed by remote sensing. Thus, only the effect that the magnetic field causes in outward flow must be deduced from the IPS LoS scintillation levels or velocity. For both the IPS-ENLIL kernel and SUSANOO-CME 3D MHD techniques, the analyses take considerably more computer resources. Additionally, the parameters fit by LoS or in situ are generally not sufficient to provide higher-resolution limitations, or continuous analyses of both the background solar wind and CMEs than the IPS kinematic model.

Analyses with more abundant LoS, especially from data at other world longitudes have been explored in Jackson et al. (2022), Iwai et al. (2022), or Fallows et al. (2022) under the auspices of the Worldwide IPS Stations (WIPSS) network (Bisi et al., 2016; Jackson et al., 2018). This would help considerably for future IPS forecast analyses, but so far, as mentioned in the introduction, data other than from ISEE have not been made available in near real time. The greatest advantage in using data from other stations, lies not so much toward providing more LoS data, but rather in filling in data gaps when an individual station has outages. Even more importantly, improvement lies in viewing the fastest outward-moving CME structures present near the Sun at times other than those of the solar transit at any one Earth longitude location.

**Figure 15:**
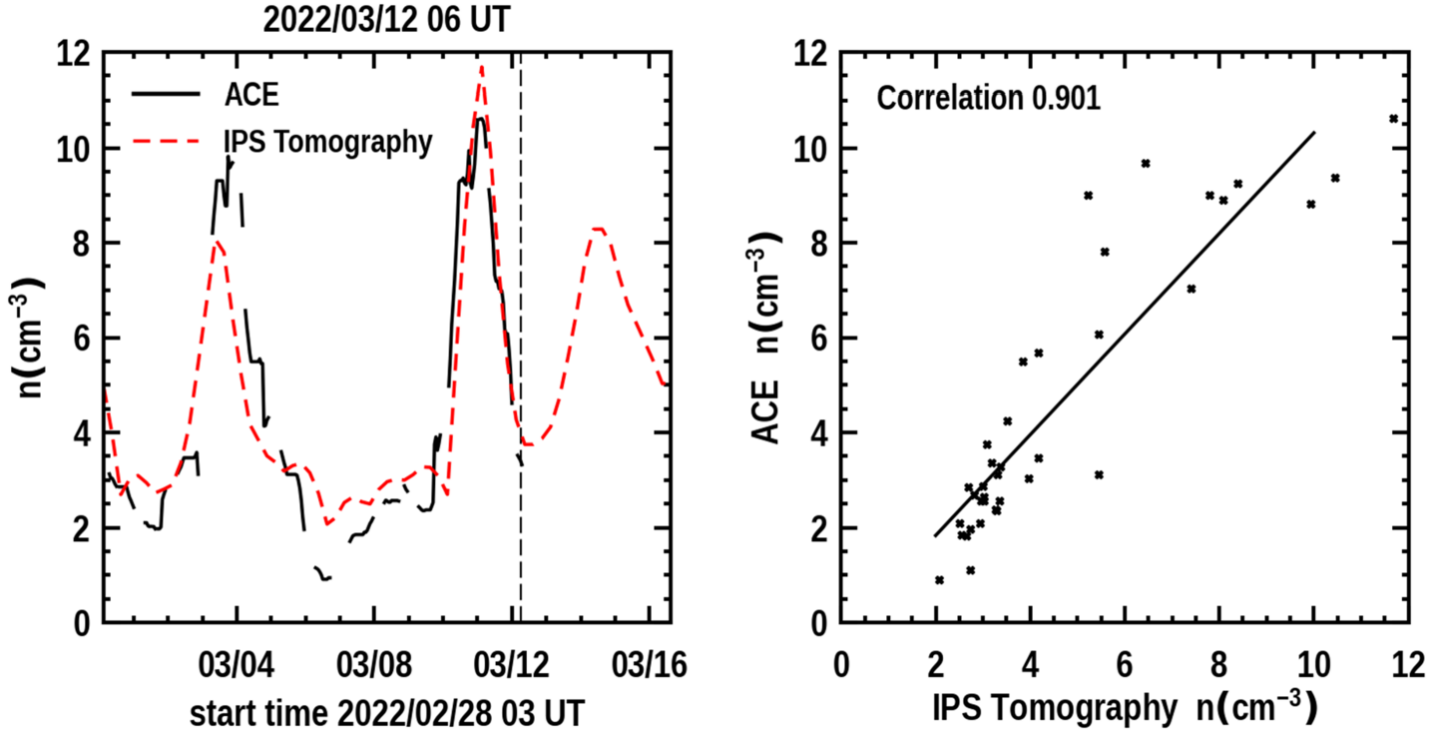
The 3D reconstructed density time series at Earth (dashed line) is shown to the left and compared with ACE in-situ density data for the same period that was available up to 6 UT March 12, 2022 at the program run time to normalize the analysis. The time at the top of the plot indicates the program forecast time at 6 UT. To the right is a Pearson’s R correlation provided for the analyses.

Forecasts from the IPS analyses can only be made after the IPS data are provided by the IPS systems that collect the data. With current internet systems the processed IPS data can usually be transmitted around the world quickly. On modern computers these data should be able to be collected from the IPS radio system and processed within a matter of minutes after they have been obtained. At the ISEE 327 MHz radio frequency, there is a near-Sun limit of ∼11.5° where strong scattering impedes measuring IPS $g$-level values accurately; IPS observations nearer the Sun to view Earth-directed CMEs are difficult to obtain from radio systems that view at higher radio frequencies. Lower-frequency phased-array systems like the Low Frequency Array (LOFAR) based at ASTRON in the Netherlands are simpler to build and maintain, and for their size can more easily view to greater elongations. However, this generally means these systems cannot look as closely to the Sun as those viewing at 327 MHz. As mentioned, the original intent of these instruments has generally been the exploration of the IPS scientific process and the long-term temporal variations of the global heliosphere. This unique scientific analysis has been well exploited by the group at ISEE, Japan. However, were the ISEE group able to provide an analysis of their data perhaps three hours following the Sun’s transit at 6 UT on 12 March, the analysis of their data would have been viewed as in Figure 15. This would have provided a 28-hour lead time for the 10 March CME from essentially the same data set. Figure 15 shows very little change in the forecast, and in this case the analysis provides a slightly better fitting prior to the forecast time as can be seen in comparison with Figure 7a.

## Conclusions

The iterative 3D reconstruction technique has been presented on UCSD webpages at: ips.ucsd.edu since the year 2000, and other space-weather groups (i.e., the NASA Goddard CCMC, Maryland; George Mason University, Virginia; ISEE, Japan; the Korean Space Weather Center; RAL Space in the UK; and the UK Met Office), have used these analyses or run them at their institutions to help provide forecasts. However, there have been few details about this forecast technique in the literature. This is mainly because the same time-dependent iterative 3D reconstruction technique works even better from a data archive where IPS observations both prior to and following the program run-time exists (as in Jackson et al., 2020, 2022, and references therein). In this article, we have kept a strict reliance on the use of the 3D reconstruction modeling from the IPS forecast technique, and the data available only prior to the programming run time that certifies and confirms the analysis results on 21 UT 12 March 2022.

This analysis shows the morphology and structure of the 10 March 2022 halo CME in low resolution as it moves outward from the Sun, and before it arrives at Earth as it was forecast. The CME presents a loop-like appearance in the different orientation cuts through the main portion of the event rotated around the Sun–Earth line. This also shows that the CME-associated material continues to flow outward over solar distances exceeding 1 AU following the CME front. These analyses also show an even more massive preceding halo CME that erupted from the Sun on 8 March but traveled more to the west of the Sun. The smoothed representations of the CME density structure give a good idea of the total extent of the material associated with the event, but this structure is shown to manifest itself in a different form at each of the in-situ monitors that the material intersects.

The CME events of 8 March and 10 March 2022 were unique in that the two interplanetary spacecraft, Solar Orbiter and BepiColombo were aligned well with both the Earth’s spacecraft and the STEREO spacecraft, respectively, so that the same events could be observed in situ by them from close solar distances. Additionally, the CME was well observed by many radio sources mapped with the ISEE system, and in many ways this was a chance occurrence that provided a good low-resolution forecast accounting of the CME morphology and its certification, especially by both the BepiColombo ESA MAG and the JAXA MEA instruments. Both the 8 March and 10 March 2022 CMEs dominated the heliospheric density along the Sun–Earth line and to the west and north of Earth, and had a lesser effect in the direction of the BepiColombo and STEREO spacecraft that were on the ecliptic plane ∼34° east of the Earth. This can be seen well in the coronagraph images and is as expected. That the 10 March CME manifestation arrived earlier at STEREO than at ACE can be accounted for by its propagation into a higher-speed background solar wind. The coronagraph observations of the 10 March CME seem to show two loops present, one to the north and another to the west. Although listed as one CME perhaps even more could be done with the 3D reconstructions to certify the extent and interaction of the total system in coordination with the existing in-situ measurements.

The different spacecraft near Earth show greatly different manifestations of the two CMEs in density, and for the IPS analyses that rely on these data sets for amplitude comparisons, this is somewhat disturbing. The current IPS 3D kinematic reconstruction analysis does not reproduce the sharp shock-density responses of these CMEs well, but neither do the different near-Earth in-situ monitors. Thus, the actual density responses and shock propagations shown by plasma-density enhancements using IPS data sets are still a problematic issue. The CME 3D reconstructed density as well as the background solar-wind density is somewhat lower in the analysis at Solar Orbiter than for the CME or the background solar-wind density at Earth measured by ACE. To provide larger values at Solar Orbiter would then not conserve mass flux from radial flow along the Earth-Sun line. Thus, the somewhat larger density that is measured by Wind spacecraft might be a more accurate determination for the solar-wind density near Earth. Since the 3D reconstruction at BepiColombo relies on the ACE calibration in Figure 12, the multiplier (1.5) used to provide a better comparison of the shielded MEA instrument electron density with a potential-field approximation subtracted might also be too low for this interval.

Similar 3D reconstruction forecasts will certainly benefit from more data, and greater longitudinal coverage from different worldwide IPS stations. Only when these analyses can put together many different longitudinal IPS stations will this enable a determination of the structure of the fastest CMEs. More stations will also enable far more CME structural changes and evolution to be observed in transit from the Sun. In the analyses here, IPS CME velocity was not present for the March CME events, and this has hampered further analysis that could have been gleaned from these CMEs globally. Velocities should be available from new IPS array systems year around and provide as many valid observations as IPS $g$-level. In addition, the greatest latency for data analysis would benefit from observations being made available as each IPS source is observed; there is little reason that the analyses could not be updated hourly if data were available. In short, many enhancements are possible for these forecast analyses; the future of these efforts is currently in progress, and these efforts deserve strong support from the heliospheric community.
